# Modified microbiology through enhanced denitrification by addition of various organic substances—temperature effect

**DOI:** 10.1007/s11356-023-26784-x

**Published:** 2023-04-06

**Authors:** Felix Ortmeyer, Marco Alexandre Guerreiro, Dominik Begerow, Andre Banning

**Affiliations:** 1 Hydrogeology Department, Ruhr-Universität Bochum, Universitätsstraße 150, 44801 Bochum, Germany; 2 Geological Survey of Denmark and Greenland, Øster Voldgade 10, 1350 Copenhagen and Universitetsbyen 81, 8000 Aarhus, Denmark; 3 Department of Evolution of Plants and Fungi, Ruhr-Universität Bochum, Universitätsstraße 150, 44801 Bochum, Germany; 4 Environmental Genomics, Christian-Albrechts University of Kiel, Am Botanischen Garten 1-9, 24118 Kiel, Germany; 5 Max Planck Institute for Evolutionary Biology, August-Thienemann-Str. 2, 24306 Plön, Germany; 6 University of Hamburg, Institute of Plant Sciences and Microbiology, Ohnhorststr. 18, 22609 Hamburg, Germany; 7 School of Biological, Earth and Environmental Sciences, University College Cork, Distillery Fields, North Mall, Cork, T23 N73K Ireland; 8 University College Cork, Environmental Research Institute, Lee Road, Cork, T23 XE10 Ireland

**Keywords:** Groundwater, Denitrification, Temperature, Organic carbon, Microbial community, Bacteria, Fungi

## Abstract

Worldwide, the environmental nitrate (NO_3_^−^) problem is increasingly coming into focus. These increases in NO_3_^−^ concentration result mainly from agricultural inputs and are further exacerbated by decreasing and finite geogenic NO_3_^−^ degradation capacity in aquifers. Thus, treatment methods are becoming more and more important. In this study, the effects of enhanced denitrification with addition of organic carbon (C) on thereby autochthonous occurring microbiology and compared at room temperature as well as 10 °C were investigated. Incubation of bacteria and fungi was carried out using natural sediments without degradation capacity and groundwater with high NO_3_^−^ concentrations. Addition of the four applied substrates (acetate, glucose, ascorbic acid, and ethanol) results in major differences in microbial community. Cooling to 10 °C changes the microbiology again. Relative abundances of bacteria are strongly influenced by temperature, which is probably the explanation for different denitrification rates. Fungi are much more sensitive to the milieu change with organic C. Different fungi taxa preferentially occur at one of the two temperature approaches. Major modifications of the microbial community are mainly observed whose denitrification rates strongly depend on the temperature effect. Therefore, we assume a temperature optimum of enhanced denitrification specific to each substrate, which is influenced by the microbiology.

## Introduction

The global problem of water pollution by nitrate (NO_3_^−^) (Almasri 2007) is increasingly recognized. The European Court of Justice has already penalized some EU member states for non-compliance with the Nitrates Directive (European Court of Justice 2010, 2018). Nitrate pollution mainly results from the intensive use of nitrogen (N) fertilizers, which are applied to increase agricultural productivity (Hosono et al. 2013). Excess N, which is not assimilated by plants, is then transported in large quantities as NO_3_^−^ to groundwater (Sebilo et al. 2013; Baram et al. 2017). Lenhart et al. (2021) modeled this transport of NO_3_^−^ through the vadose zone. Due to climate change and a resulting decrease in water resources in many regions, further increases in NO_3_^−^ concentrations are expected in the future (Fleck et al. 2017; Ortmeyer et al. 2021a). The World Health Organization (WHO 2011) as well as the European Drinking Water Directive (98/83/EC 1998) sets the limit for NO_3_^−^ in drinking water at 50 mg/L because high NO_3_^−^ intakes can cause methemoglobinemia in infants up to 6 months of age (Ward et al. 2018). Besides the risk for infants and the unborn, there are also strong indications for elevated cancer risks for adults (Inoue-Choi et al. 2015; Espejo-Herrera et al. 2016; Jones et al. 2017; Schullehner et al. 2018). In addition, the input of N can lead to eutrophication in the environment, which eventually threatens aquatic fauna (Dodds and Smith 2016).

Nevertheless, NO_3_^−^ can be reduced in many aquifers via redox reactions with sulfide minerals, organic carbon (C), and iron (Fe(II) phases (Rivett et al. 2008). When these redox partners are available, they represent the aquifer’s NO_3_^−^ reduction capacity. However, when NO_3_^−^ is reduced, these capacities continue to decrease and are finite (Knowles 1982). Therefore, there are some approaches to minimize NO_3_^−^ input to groundwater. For example, there are collaborations with the farming industry to reduce the amount of fertilizer, to fertilize only during certain periods, and to increase the efficiency of N uptake by plants (Cameron et al. 2013; Eulenstein et al. 2017). However, an example from Denmark shows that reducing fertilizer inputs too much can also cause problems. A worrying decrease in protein levels in grain crops was shown by a long-term study (Styczen et al. 2020). Thus, other methods are also needed to mitigate NO_3_^−^ concentrations in groundwater. Bioremediation, in which sites polluted with NO_3_^−^ compounds are decontaminated by microorganisms (e.g., bacteria), is a promising method. This method can occur naturally, but it can also be promoted by biostimulation. This technique modifies the environmental conditions by the addition of external carbon sources (e.g., ethanol or acetic acid), to enhance the activity of microorganisms that can use NO_3_^−^ compounds. Biostimulation is a promising method to decontaminate groundwater polluted by NO_3_^−^ (Azubuike et al. 2016). Biological denitrification enhanced by the addition of external C is one of the best known and most effective treatment methods for nitrate-polluted groundwater (e.g., Khan and Spalding 2004; Vidal-Gavilan et al. 2013; Ortmeyer et al. 2021b). A great advantage is the selective NO_3_^−^ removal. Denitrification requires an electron donor, which is oxidized while NO_3_^−^ is eventually reduced to environmentally harmless N_2_ gas. The general reaction sequence is as follows: NO_3_^−^  → NO_2_^−^  → NO → N_2_O → N_2_ (Knowles 1982). For example, Carrey et al. (2014) investigated the reduction potential by adding the electron donor glucose. Ge et al. (2012) used methanol, acetate, and glucose for enhanced denitrification. In addition, the reduction potential of more complex and especially non-liquid materials such as pine bar or sawdust was investigated (Schipper and Vojvodic 2000; Trois et al. 2010). On the other hand, liquid C compounds can be more readily introduced into the aquifer, so the NO_3_^−^ reduction potential of waste products from wine as well as whey production is also being investigated (Carrey et al. 2018; Margalef-Marti et al. 2019).

In addition to a required electron donor, denitrification in the environment usually occurs when microorganisms catalyze the reaction (Korom 1992). Denitrifiers account for up to 5% of the total soil microbial community (Philippot et al. 2007; Henry et al. 2004). These are not only mostly bacteria, but also fungi which can contribute up to 18% to denitrification in soil (Herold et al. 2012). Nevertheless, fungi are often considerable N_2_O producers in soils (Laughlin et al. 2009; Maeda et al. 2015). Nitrate reduction also occurs in the soil under near-anoxia conditions. In the study by Liu et al. (2019), the genera Pseudomonas, Dechloromonas, and Herbaspirillum were identified as dominant, active soil denitrifiers. Philippot and Hallin (2005) discuss the understanding of the relationship between community structure and activity of microbiology. The molecular characteristics and functional distinction between bacterial NO_3_^−^ reductases are presented by Moreno-Vivián et al. (1999).

When investigating the efficiency of biological denitrification, even the addition of microorganisms is applied (Schroeder et al. 2020). Costa et al. (2018) used microorganisms previously obtained from bamboo biomass in shaking experiments. Further studies of the vadose zone show that organic C is the limiting factor, not the lack of denitrifiers (Chen et al. 2018). Furthermore, external C addition affects bacterial community composition (Hellman et al. 2019). A study of sedge, barley straw, and pine woodchips reactors for NO_3_^−^ removal shows different bacterial communities (Hellman et al. 2021). The addition of hydrogen likewise influences the composition of the microbiology. Duffner et al. (2021) study denitrification by addition of hydrogen (H_2_) and identify the microbiology dominant for hydrogenotrophic NO_3_^−^ degradation. Vidal-Gavilan et al. (2014) reported that the selection of the specific electron donor and the feeding strategy play an important role in enhanced denitrification. Another study with an in situ addition of an electron donor where a natural microbial community formed subsequently showed a 6-week ability to continue NO_3_^−^ reduction even in the prolonged absence of the electron donor ethanol (Paradis et al. 2021). Canion et al. (2014) show that the microbial community changes during heating experiments for denitrification. Liao et al. (2018) demonstrate the change in microbiology by temperature based on altered abundance of denitrification function genes.

Ortmeyer et al. (2021b) investigated the influence of temperature on enhanced denitrification. Four pure organic substrates (acetate, glucose, ascorbic acid, ethanol) were applied, which were investigated under realistic conditions on a laboratory scale in column experiments. In contrast to many previously described research studies, it was demonstrated that the reaction kinetics of enhanced denitrification do not always increase with increasing temperature. Thus, more effective NO_3_^−^ reduction occurs with the addition of ethanol at 10 °C than at room temperature. In this study, the change and consequently the occurrence in the microbial community induced by enhanced denitrification by the four different substrates and the influence of temperature (typical Central European groundwater temperature of 10 °C and room temperature of 21.5 °C) are investigated. The microbial communities are evaluated in detail regarding bacteria and fungi. The bacterial and fungal communities are compared with respect to preferred added C and temperature. In this context, it is important to investigate fungi as well as bacteria, even if fungi play only a minor role in denitrification, to gain an understanding of the entire complexity of the processes. Thus, this multidisciplinary study aims at contributing to the deeper understanding of the biohydrogeochemical interplay of natural and anthropogenic parameters controlling one of the most severe groundwater and drinking water quality issues of our time.

## Materials and methods

For analysis of the microbial community, shaking tests were performed. 15 mL falcon tubes were filled with 6.2 g sediment. Relatively pure, medium sands of the Haltern Fm. (Upper Cretaceous, northwestern Germany) were used. The sediment has almost no geogenic NO_3_^−^ degradation capacity (sulfide-sulfur < 0.01 wt%, organic C 0.06 wt%, CS, G4 Icarus, Bruker, Ruhr-University Bochum) (Ortmeyer et al. 2021c), likewise a low reduction potential due to iron. Fe:water: < 0.1 mg/L and sediment: 1.14 wt%, in oxidized Haltern Fm. Fe is present as (hydr)oxide (Banning et al. 2009, 2013), because it is almost completely oxidized. The 15-mL falcon tubes were then filled with groundwater which had NO_3_^−^ concentrations of 200 to 250 mg/L. Natural groundwater was obtained from a piezometer in the Haltern Fm (Ortmeyer et al. 2021b). Consequently, the water corresponds hydrogeochemically to the sediment used. Falcon tubes were filled without air inclusion. In addition, each groundwater contained one of the four different selected C: acetate, glucose, ascorbic acid (manufacturer: CHEMSOLUTE), and ethanol (manufacturer: VWR) to initiate a heterotrophic denitrification. The individual substrates were added in three concentration steps, according to Ortmeyer et al. (2021b). In each of the column experiments in Ortmeyer et al. (2021b), with a water volume of 3.74 L, substances were added in the steps 2.5, 5, and 10 mmol of the four organic carbon (*C*_org_). Concentrations of *C*_org_ in the 15-mL falcon tubes were adjusted to the concentrations from the column experiments. For a better overview and comparability, the values 2.5, 5, and 10 mmol are used here as well. As a control, an additional shaking experiment was carried out with the same sediment as described above and pure groundwater, without *C*_org_ addition. The shaking tests were performed at room temperature (21.5 ± 2 °C) and at 10 °C the typical groundwater temperature in Germany. To assure replicability of the experiment, all shaking tests were conducted in triplicate. This results in a total of 78 shaking tests (four different organic C, three concentration steps, three control samples, and each at two temperature approaches).

To simulate flow, the overhead shaker was set to the lowest speed. Since the column experiments in Ortmeyer et al. (2021b) identified the first week after addition of the substrates as the most important for denitrification, the shaking tests to identify the microbial community also ran for 1 week. Shaking tests at room temperature were performed in a darkened room, which was tempered to 21.5 °C. For 10 °C, the overhead shaker was placed in a refrigerator, tempered to 10 °C.

To characterize the bacterial community as well as fungi communities in the water samples, 7 mL of the supernatant water was centrifuged in several steps in a 500 µL, 0.2-µm filter mini column for 2 min at 13,000 rpm. The filters containing microbial biomass were transferred to lysis tubes E (MP Biomedicals), and then, 400 μL of SLS lysis buffer was added. This was followed by mechanical disruption (3 × 6 m/s, 45 s) using a FastPrep-24™ instrument (MP Biomedicals). In the next step, 20 µL of proteinase K was added and the reaction was thoroughly mixed. According to Graupner et al. (2017) and the manufacturer’s instructions, microbial DNA was then extracted using the my-Budget DNA Mini Kit (BioBudget Technologies, Krefeld, Germany). The isolated DNA was stored at − 20 °C after extraction. The 16S (5′-CCTACGGGNGGCWGCAG-3′ and 5′-GACTACHVGGGTATCTAATCC-3′) and ITS (5′-CGCTTATTGATATGCTTAAGT-3′ and 5′-GTGARTCATCGARTCTTTG-3′) amplicon libraries were prepared by the German Institute of Mycology (Bayreuth, Germany). Sequencing was performed by the sequencing service of the Faculty of Biology at LMU Munich, using an Illumina MiSeq® sequencer (2 × 250 bp paired end sequencing). According to Röhl et al. (2017), the sequence reads were processed. These are available from the European Nucleotide Archive (http://www.ebi.ac.uk/ena/) under study PRJEB42532 and PRJEB52267. Bacterial OTUs were taxonomically classified by using the SILVA database version 138.1 and the RDP classifier version 2.11 (16S rRNA training set no. 18 07/2020), while for fungal OTUs, the taxonomic affiliation was based on UNITE database version 8.2 (Kõljalg et al. 2013). Samples that yielded a low amount of sequencing data (< 20 reads) were excluded from further analyses. The reasons behind this low amount of data are unknown and could be either biological and/or technical.

## Results

Denitrification was induced with the addition of four organic substrates. The results of the denitrification evaluated in Ortmeyer et al. (2021b) show a different NO_3_^−^ reduction depending on the added C, but also a difference through the two temperature approaches of the same C. The occurrence of bacteria and fungi also differ among the individual substrates and here too in the two temperature approaches.

In this study, a more detailed evaluation of microbiological results is carried out, with data for bacteria, fungi, and finally their joint occurrence being presented.

### Bacterial communities

Amplicon sequencing yielded 587,377 quality-filtered 16S reads, which clustered into 37 bacterial OTUs. Sequencing did not detect any barcodes derived from Archaea, so it can be assumed that Archaea were rare in this experimental setup and were below the detection limit. Taxonomic classification revealed that eight bacterial phyla and one unidentified phylum were present in the samples. The relative abundance of phyla was previously published in Ortmeyer et al. (2021b). The microbial community was strongly affected by the addition of the different C as well as the two temperature approaches compared to the control sample. Actinobacteria and Proteobacteria dominated the samples at 21.5 °C incubation. At the incubation of 10 °C, Actinobacteria dominated most. The temperature effect was particularly evident in the samples with the addition of ethanol. At 21.5 °C, Proteobacteria were dominant (98%), whereas at 10 °C, Actinobacteria were dominant (89 to 93%).

**Fig. 1:**
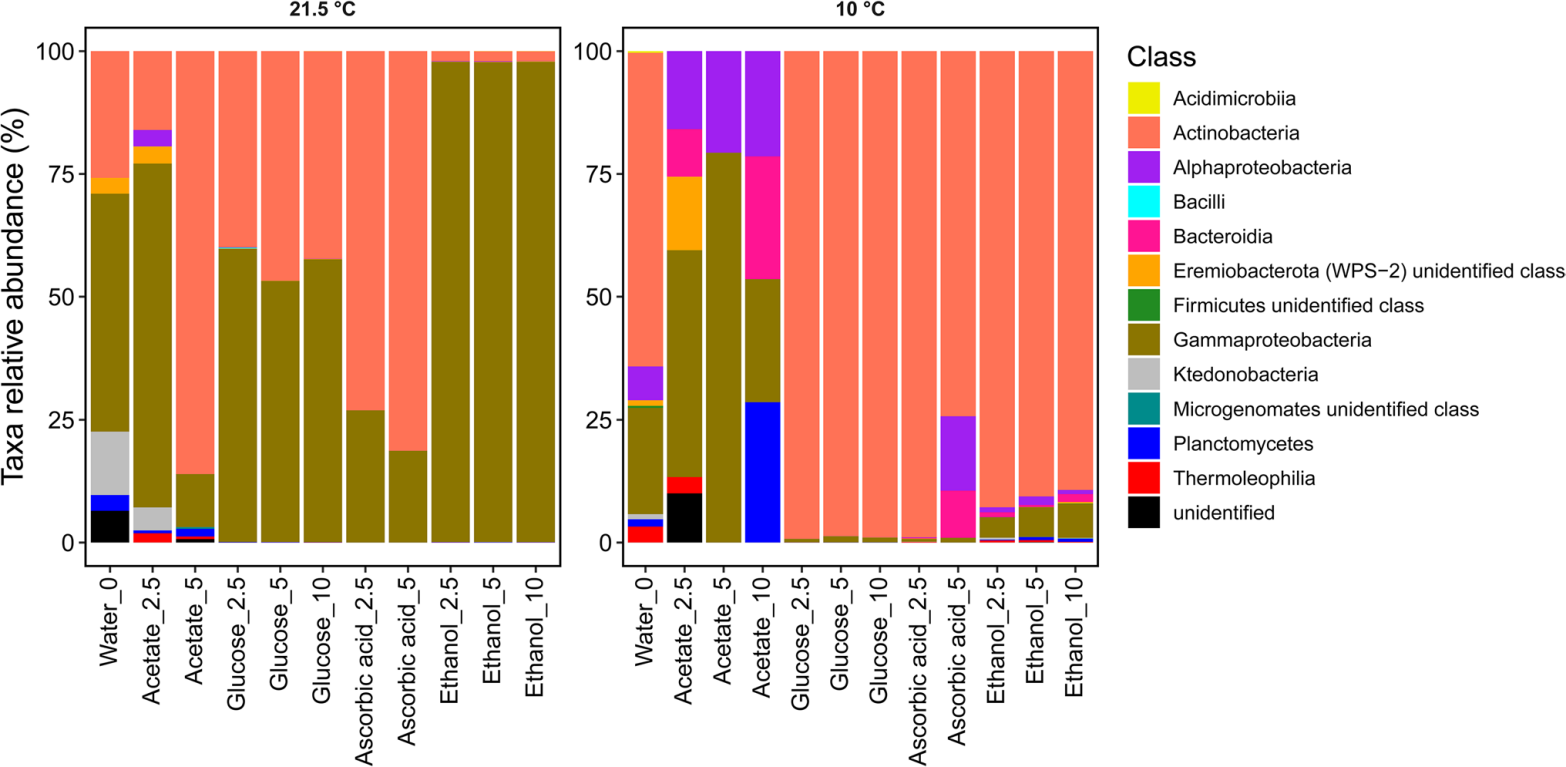
Relative abundances of bacterial classes in samples containing different organic C additions (concentration steps 2.5; 5 and 10 mmol), incubated at room temperature (21.5 °C) or at 10 °C, and a control sample (sediment + water) without *C*_org_ addition

Figure 1 shows the composition of the microbiological community in more detail at the class level. When incubated at 21.5 °C, it is noticeable that there was a similar distribution as at the level of phyla. Thus, several classes of a phylum are not included, rather only one specific class from the phyla occurs in most cases. The samples with addition of glucose, incubated at 21.5 °C, were dominated by Gammaproteobacteria (56 to 60%) and Actinobacteria (40 to 44%). However, when these samples were incubated at 10 °C, the samples were dominated by Actinobacteria (99%). With the addition of ethanol, the change between the two temperature approaches is even greater. At 21.5 °C, Gammaproteobacteria were 98% dominant. At 10 °C incubation, Actinobacteria were dominant by 88 to 93%. Samples with the addition of acetate and incubated at 21.5 °C showed different dominant bacterial classes depending on the amount of substance added. When 2.5 mmol acetate was added, Gammaproteobacteria (70%) were dominant, and when 5 mmol acetate was added, Actinobacteria were dominant (85%). In addition, it is noticeable that several classes of a phylum appear at the incubation of 10 °C when acetate is added. Samples with addition of 5 mmol ascorbic acid were dominated by Actinobacteria (81 and 74%) in both temperature approaches. In addition, Proteobacteria appeared. However, a closer look at the level of the classes shows that with an incubation of 21.5 °C, Gammaproteobacteria (19%) appeared and with an incubation of 10 °C, Alphaproteobacteria (15%) occurred.

**Fig. 2:**
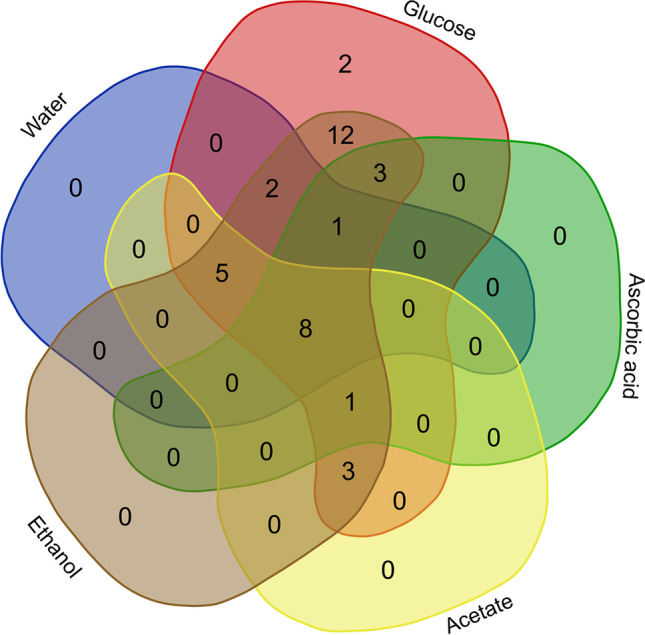
Occurrence of bacterial species divided into preferred organic substrates as well as the control sample (sediment + water without *C*_org_ addition)

Figure 2 shows, subdivided according to the individual C, whether the bacterial species occur individually or together with other bacteria, due to the substrate addition. Thus, the bacteria usually do not only occur through the addition of one substrate; they are often also found through the addition of the other applied organic C. Only two species of the classes Gammaproteobacteria and Bacilli appear due to the addition of glucose. Another 12 species (5 species belonging to Gammaproteobacteria, 4 Actinobacteria, 2 Alphaproteobacteria, 1 Planctomycetes) occur only by the addition of glucose and ethanol. In contrast, 8 species (3 species belonging to Gammaproteobacteria, 1 Actinobacteria, 1 Alphaproteobacteria, 1 Ktedonobacteria, 1 Thermoleophilia, 1 Eremiobacterota (phylum: class unidentified)) can be detected by the addition of all substrates. All remaining species occur through at least three substrates (or control). Thus, the samples with the addition of acetate, ascorbic acid, and the control sample have no species that occur only through one or two substrates.

**Fig. 3:**
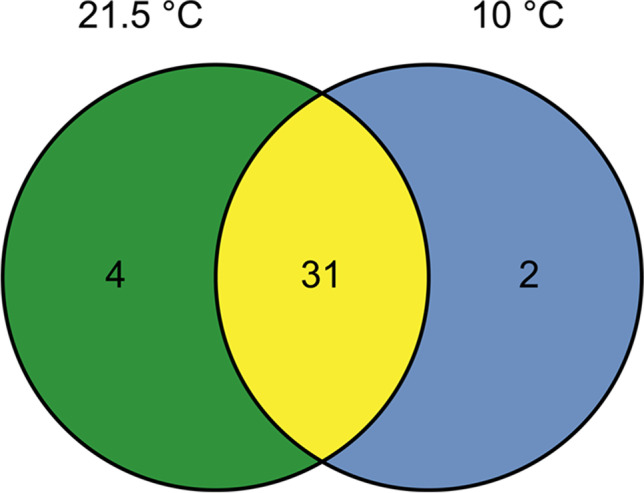
Occurrence of bacterial species divided into preferred temperature: 21.5 °C: green, both temperatures: yellow, 10 °C: blue

A similar observation can be made when investigating the preferred temperature. Most species can be detected at both temperature approaches (Fig. 3). Only four species occur exclusively at 21.5 °C. These are from the classes Gammaproteobacteria and Bacilli. In contrast, two species occur exclusively at the typical groundwater temperature of 10 °C. Thus, the classes Alphaproteobacteria and Bacteroidia are detected at this temperature.

### Fungal communities

**Fig. 4:**
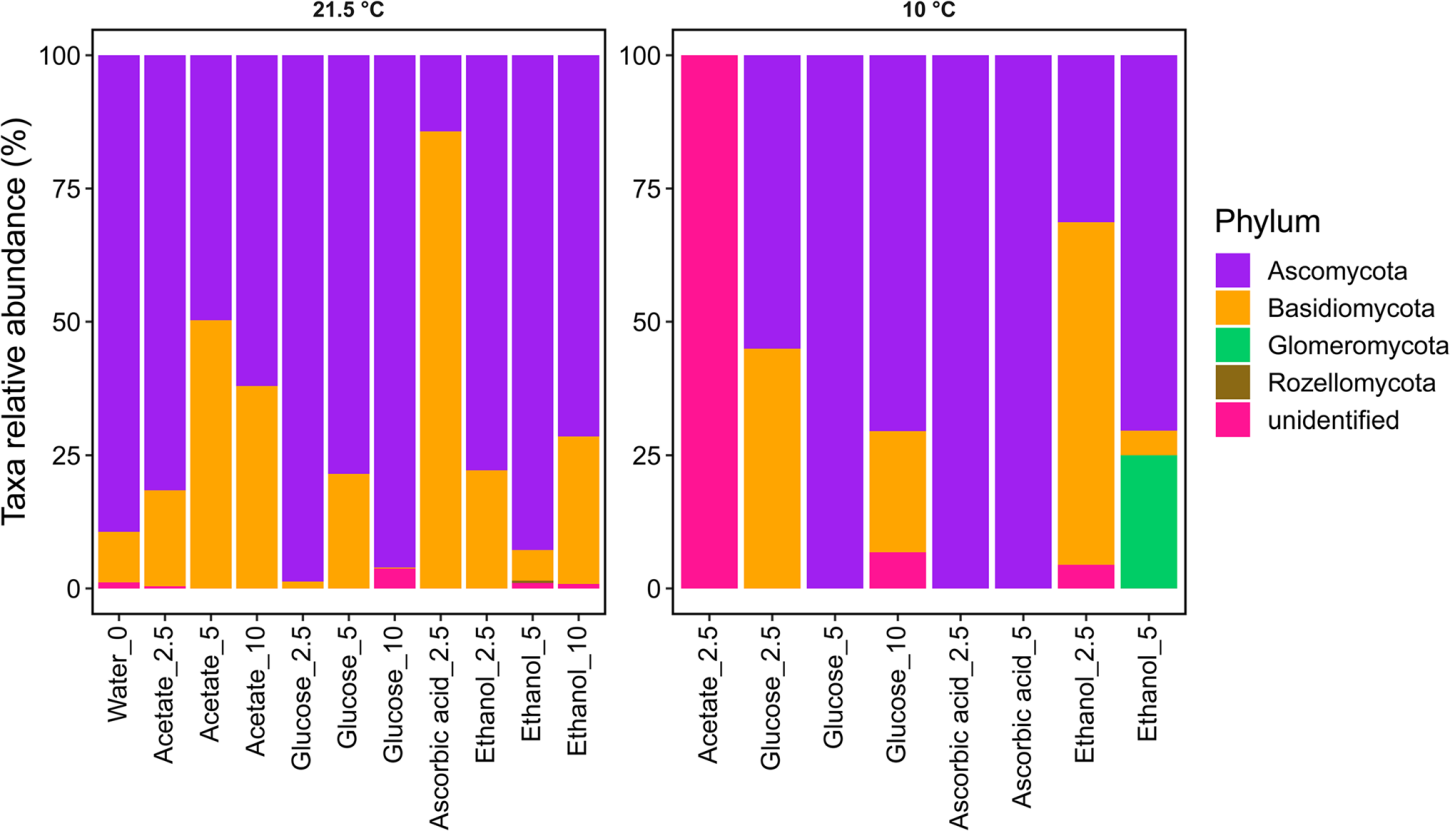
Relative abundances of fungus phyla in samples containing different organic C additions (concentration steps 2.5; 5 and 10 mmol), incubated at room temperature (21.5 °C) or at 10 °C, and a control sample (sediment + water) without *C*_org_ addition

Fungal amplicon sequencing yielded 40,299 quality-filtered ITS1 reads, which clustered into 93 fungal OTUs. Figure 4 shows the relative abundance of these fungal phyla. With the taxonomic classification, the four phyla Ascomycota, Basidiomycota, Glomeromycota, and Rozellomycota could be identified. Additionally, one unidentified phylum was found in the samples. Control samples without *C*_org_ were dominated by Ascomycota (89%). Basidiomycota had the second highest relative abundance at 10%. The microbiological community of fungi was slightly less affected by addition of organic C at the phyla level than bacteria. Ascomycota were also dominant in the samples with addition of organic C incubated at 21.5 °C. In samples with addition of acetate, Ascomycota were present between 50 and 81%, followed by Basidiomycota between 18 and 50%. The relative abundance of Ascomycota in the samples with addition of glucose was 79 to 99%. In samples with ethanol, Ascomycota occurred between 72 and 93%. The only exception was the addition of ascorbic acid. Here, Basidiomycota dominated the samples with 86%. Ascomycota were present in only 15%.

When incubated at 10 °C, compared to room temperature (21.5 °C), the microbial communities of the fungi were greatly affected and altered. In samples with the addition of acetate, 100% of the undefined phylum occurred at 10 °C incubation, which was just 0.4% at 21.5 °C. Ascomycota (100%) were exclusively present in the ascorbic acid samples at 10 °C incubation. Glucose samples incubated at 10 °C showed a greater variation in the occurrence of Ascomycota (55 to 100%). The dominance of Ascomycota in samples with ethanol incubated at 10 °C (31 to 70%) decreased and also showed greater variation in occurrence. At 2.5 mmol ethanol, the relative proportion of Basidiomycota increased to 64%, with a decrease in relative proportion in samples with 5 mmol ethanol (5%) and the appearance of Glomeromycota (25%).

**Fig. 5:**
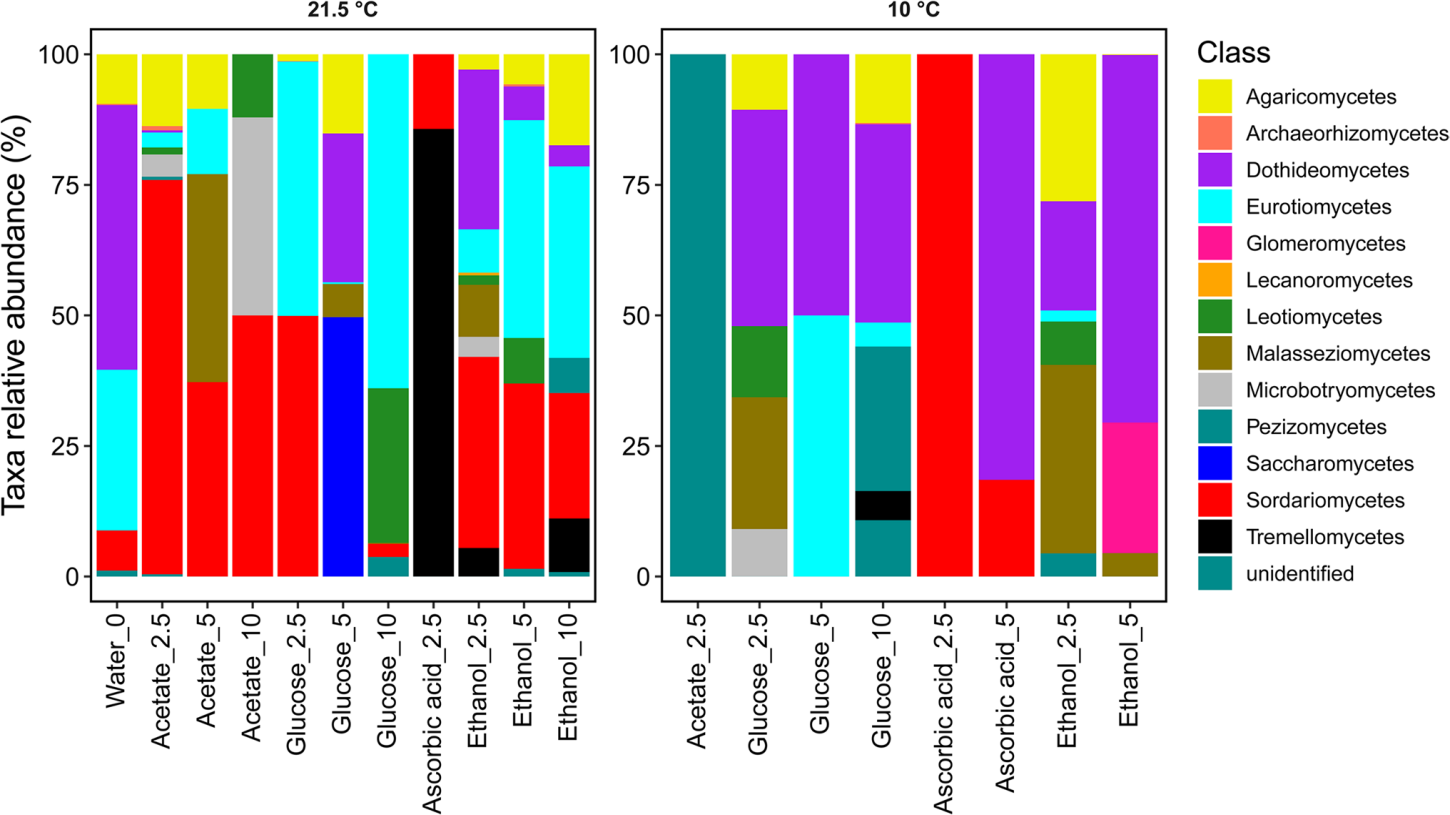
Relative abundances of fungus classes in samples containing different organic C additions (concentration steps 2.5; 5 and 10 mmol), incubated at room temperature (21.5 °C) or at 10 °C, and a control sample (sediment + water) without *C*_org_ addition

In contrast to the bacteria, considerably more classes of a phylum occurred in the fungi and relative abundances of occurrence were divided into numerous classes (Fig. 5). At the class level, the addition of organic C incubated at 21.5 °C reveals a considerably greater change in the relative abundances of the fungi than at phyla level. In the control sample (water only, no C addition), Dothideomycetes (51%) and Eurotiomycetes (31%) dominated the abundance. Acetate samples incubated at 21.5 °C were also dominated by fungi of the phyla Ascomycota, as described. However, at the class level, the fungi that appeared differed clearly from the control sample. The class Sordariomycetes was dominant (37 to 76%). Samples with the addition of glucose differed in all three concentration levels, although they showed a relatively uniform occurrence of Ascomycota at the phyla level. The samples with the addition of glucose exhibited the following relative abundances: glucose 2.5 mmol (Sordariomycetes 50%, Eurotiomycetes 49%), glucose 5 mmol (Saccharomycetes 50%, Dothideomycetes 29%), and glucose 10 mmol (Eurotiomycetes 64%, Leotiomycetes 30%). With the addition of ascorbic acid, the sample had only one class per phylum and were dominated by Tremellomycetes (86%). Ethanol samples, similar to the acetate samples, had many different phylum Ascomycota classes. With the addition of 2.5 mmol, Sordariomycetes (37%) and Dothideomycetes (31%) were dominant. At 5 and 10 mmol ethanol addition, Eurotiomycetes (37 to 42%) and Sordariomycetes (24 to 36%) were dominant.

Incubation at 10 °C resulted in considerably less different classes than the 21.5 °C incubation. In addition, the microbial community changed greatly in composition. Thus, in samples containing 2.5 mmol acetate, 100% of the unidentified class occurred. Dothideomycetes (38 to 50%) appeared dominant in glucose samples incubated at 10 °C, which were previously dominant in the control sample incubated at 21.5 °C. Similarly, a clear change in the microbial community occurred in the samples incubated with 2.5 mmol ascorbic acid. At 21.5 °C, the samples were dominated by Tremellomycetes, while at 10 °C, it was Sordariomycetes (100%). The phylum Ascomycota in ascorbic acid samples with 5 mmol addition included the classes Dothideomycetes (82%) and Sordariomycetes (19%). With 2.5 mmol ethanol, several classes were present even at 10 °C incubation. Compared to incubation at 21.5 °C, the relative proportion of Malasseziomycetes (36%) and Agaricomycetes (28%) increased considerably, while Dothideomycetes decreased to 21%. Dothideomycetes, on the other hand, was dominant (70%) in samples with 5 mmol ethanol at 10 °C incubation. It was furthermore the only sample in which Glomeromycetes was present to a greater relative proportion (25%).

**Fig. 6:**
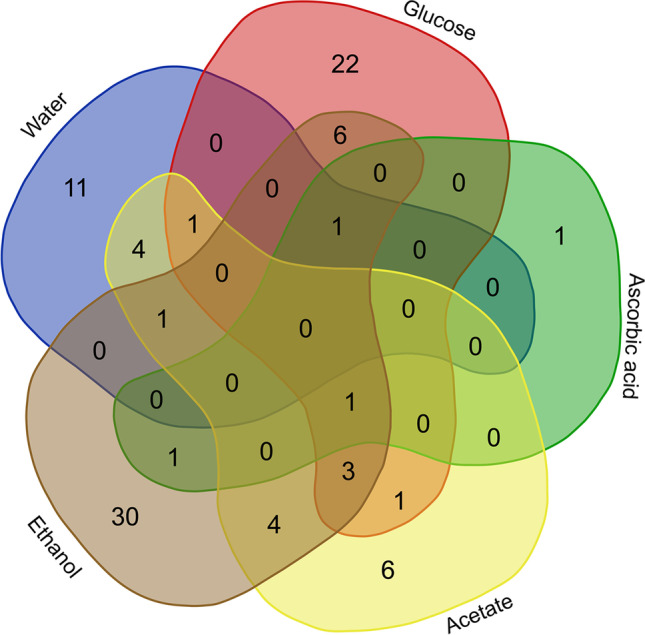
Occurrence of fungal species divided into preferred organic substrates as well as the control sample (sediment + water without *C*_org_ addition)

Contrary to the bacteria, fungal species mainly appear only by the addition of a specific substrate and are very rarely detected by the addition of the other substrates or the control (Fig. 6). No species occurs by addition at all organic C. Only two species occur in three substrates and water control. By adding ethanol, 30 species can be found only in these samples. Glucose also contains 22 species that can only be found in the samples with glucose. However, six species can be detected by the addition of ethanol and glucose. Thus, the samples with the application of ethanol and glucose show the most species in total. Even the control samples, which contains only water, exhibits 11 species that can only be found in these samples. Acetate and ascorbic acid show only a few fungal species (six and one, respectively), which could only be detected by its addition. The addition of ascorbic acid resulted in the detection of a total of only three fungal species.

**Fig. 7:**
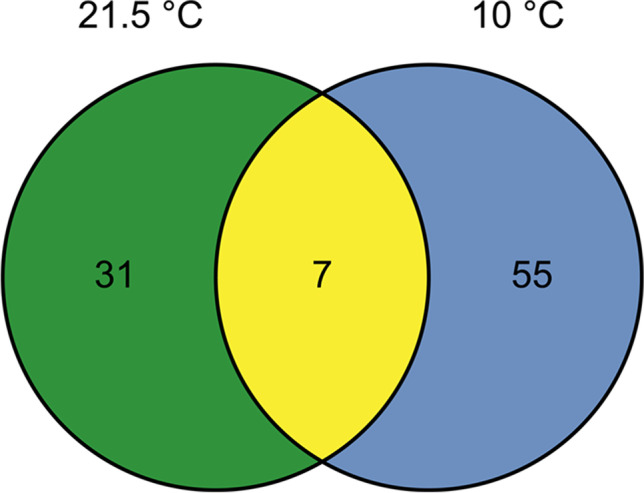
Occurrence of fungal species divided into preferred temperature: 21.5 °C: green, both temperatures: yellow, 10 °C: blue

Similar to the investigation of the preferred C, the fungal species mainly prefer one of the two temperature approaches (Fig. 7). Thus, 31 species occur at 21.5 °C. At the typical groundwater temperature of 10 °C, 55 fungal species can be detected. Only seven species can be detected in both temperature approaches.

### Comparison between bacteria and fungi

**Fig. 8:**
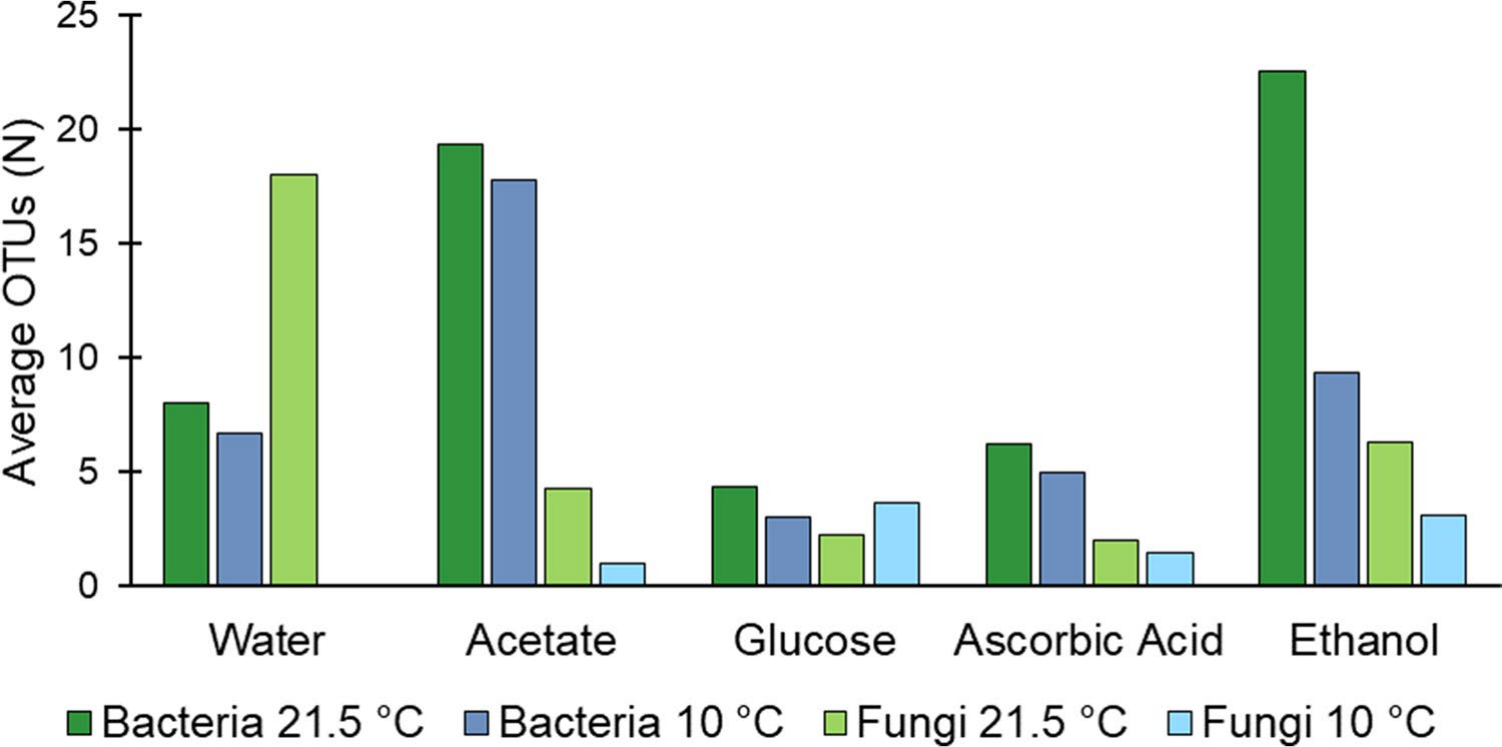
Occurrence of average OTUs of bacteria and fungi at 21.5 and 10 °C subdivided by the applied organic substrates

Figure 8 shows the average number of different bacterial and fungal species (dark blue and dark green: bacteria; light blue and light green: fungi). A distinction is only made between the four substrates (and water control) and the two temperature approaches (21.5 and 10 °C). The three concentration steps are not considered here.

Thus, it becomes clear that temperature rarely plays a role in the occurrence of the average number of species of bacteria. Furthermore, the difference in the number of species between the two temperature approaches is usually only slightly smaller at 10 °C. However, for ethanol, the average species number of bacterial species at room temperature is considerably larger (22.6). At 10 °C, the average species number is 9.3. Due to the addition of glucose and ascorbic acid, few bacterial species appear on average (4.3 and 3.0, 6.2 and 5.0, respectively). These are also fewer species than in the control sample. With the addition of acetate, an average of 19.3 species are detected at room temperature and 17.8 species at 10 °C.

In the control sample, considerably more fungal than bacterial species are detected on average at 21.5 °C (18.0). However, in the control samples incubated at 10 °C, no fungal species are present. Even in the samples with the addition of the four substrates, fungal species numbers are on average very low at 10 °C. However, the average species numbers are also very low at room temperature and are far below the species numbers of the control sample. Ethanol has the highest average number of species at both temperatures (6.3 and 3.1, respectively). Ascorbic acid has the lowest average number of species at 21.5 °C (2.0) and acetates at 10 °C (1.5).

## Discussion

The composition of the occurring bacterial and fungal communities differs strongly when applying the four organic substrates used in this study (acetate, glucose, ascorbic acid, and ethanol) as well as the two temperatures (room temperature of 21.5 °C and 10 °C, the typical groundwater temperature in Germany). Similarly, the denitrification rates and efficiency differ greatly among the organic C applied and likewise between the two temperature approaches (Ortmeyer et al. 2021b). Since the sediments used do not contain any NO_3_^−^ degradation capacity, it can be excluded that this change in the microbial community was caused by a geogenic degradation capacity. Thus, we conclude that these modifications result from the applied substrates as well as from the influence of the two temperature approaches. Bacteria and fungi are compared at class level, since most OTUs could not be accurately classified at lower taxonomic levels, such as genus. This is due to a lack of reference barcode data in databases for accurate classification. Given the very selective conditions used in this study (temperature and C sources), it is likely that the detected microorganisms are understudied and lacking in reference databases. Further studies should additionally investigate the change in microbial community after a longer period of C addition. Since this study focuses on the change in microbiology after 1 week, the period in which the highest denitrification rate was observed. So it can be compared if there are any further changes by an addition over a long period.

With the applied four organic substrates, Actinobacteria and Gammaproteobacteria were dominant at room temperature. At 10 °C, Actinobacteria were particularly dominant. Ginigen et al. (2013) also describe reduced microbial diversity in NO_3_^−^ containing artificial recharged groundwater; their geochemical data indicate autotrophic denitrification. Detailed evaluation of the bacteria performed in this study shows that the phyla identified in the previous study (Ortmeyer et al. 2021b) were mostly present in only one class and not subdivided into many different classes. By addition of the substrates in each case, specific bacteria of the phyla seem to appear. In contrast, the individual species of the bacteria often did not only occur with the addition of a specific substrate but can be detected after the addition of several substrates. Consequently, this allows the conclusion that the occurring bacteria all frequently occur during enhanced denitrification with the addition of organic C but react differently to the individual substrates and catalyze denitrification with different efficiency. A comparison with bioreactors, in which sedge, straw, and woodchips was used likewise at 10 °C, showed that different bacterial communities developed in the three types of substrates as well (Hellman et al. 2021).

Furthermore, the general occurrence of bacterial species is not temperature dependent, as most species were detectable at both temperature approaches. As an exemption, Brevundimonas and Hydrotalea of the classes Alphaproteobacteria and Bacteroidia, respectively, occurred only at 10 °C. Whereas Burkholderia-Caballeronia-Paraburkholderia (Gammaproteobacteria) and Bacillus (Bacilli) occurred only at 21.5 °C. Chen et al. (2018) also found an accumulation of Bacillus in their experiments on denitrification at 18 °C in the vadose zone. The general temperature-independent occurrence of bacterial species is contrasted by the changes in relative abundances of bacteria and thus, above all, the different denitrification rates as well as different efficiency of the substrates at room and groundwater temperature of the preceding study (Ortmeyer et al. 2021b). Consequently, the bacteria generally occur at both temperatures, but are considerably more effective at one of the two temperature approaches. However, due to the complexity of the process, we do not assume increasing denitrification rates and efficiencies with increasing or decreasing temperatures, but rather temperature optima depending on the dominance of particular bacterial species (Ortmeyer et al. 2021b).

A comparison with a study by Carlson et al. (2018) examining a contaminated aquifer indicates that temperature exerts less selective pressure on microbiology than trace elements, such as Cd, Co, Ni, U, and Zn. While the individual trace elements have different strong influences on microbiology.

A more detailed evaluation of the individual substrates shows the presence of Burkholderiaceae and Bacillaceae of the classes Gammaproteobacteria and Bacilli only in the incubation with glucose. Gammaproteobacteria were present at > 50% at room temperature in all three concentration approaches of glucose addition. At 10 °C, almost no Gammaproteobacteria occurred by glucose addition (≤ 1%). This may explain the observation obtained in Ortmeyer et al. (2021b) where the strongest NO_3_^−^ reduction of the study occurs at room temperature with glucose, but the reduction rates at 10 °C were considerably lower. Additionally, the average species number with glucose is almost the same between room temperature and 10 °C (cf. Figure 8), although the efficiency of denitrification is very dependent on temperature. Overall, no direct relationship between the number of OTUs and denitrification efficiency was found in the systems with different substrates at different temperatures. Under restrictive conditions, species richness is likely to be lower. Additionally, some OTUs might be present in the system independently of their denitrification ability. The most effective NO_3_^−^ reduction at 10 °C was detected in Ortmeyer et al. (2021b) with the addition of ethanol. Here, Actinobacteria were particularly dominant at 10 °C in all three concentration approaches (88.3 to 92.8%). These were even more dominant in the samples with glucose addition (98.8 to 99.3%). Nevertheless, more effective denitrification occurs with ethanol addition. Furthermore, it is observed that 12 bacterial species belonging to the classes Proteobacteria, Actinobacteria, and Planctomycetota occur only with the addition of glucose and ethanol. Therefore, the question arises whether a combined addition of ethanol followed by glucose can increase the efficiency of glucose at 10 °C and whether this efficiency is related to the different optimal growth temperatures and C sources of the different species. Thus, possibly a combination of two substrates could increase the efficiency at groundwater temperatures to a similar efficiency as at room temperature if the microbial community was previously stimulated with a substrate that is very effective at low temperatures. Schroeder et al. (2020) first used ethanol and then replaced this with the substrate glycerol. Their results show an efficient NO_3_^−^ reduction with glycerol after ethanol replacement. However, these experiments are conducted at 28 °C, so the question is whether this can also be transferred to a groundwater system at 10 °C. Also, it is not uncommon to isolate bacteria from other media, for example, bamboo biomass (Costa et al. 2018) but also groundwater (Carlson et al. 2018). Thus, there is also a potential for increasing the reduction potential of glucose at 10 °C by the addition of previously isolated bacteria.

In contrast to bacteria, the evaluation of fungi in this study shows that the occurring phyla can be classified into numerous different fungal classes. However, the occurrence of fungal species is mainly caused only by one specific substrate. But the average number of fungal OTUs present is greatly reduced by the addition of the substrates. Thus, incubation with added organic C causes some fungal species to die or be supplanted. Similarly, the occurrence of fungal species is temperature dependent. Most species appear at either 21.5 or 10 °C. The average OTUs illustrate that the fungal species in the control sample suffer from the low temperature (10 °C). In the samples with addition of the four different substrates, average OTUs are also low, but specific fungal species may occur due to the organic C present, which is illustrated in Fig. 6.

The more effective NO_3_^−^ reduction with the addition of ethanol at 10 °C described in Ortmeyer et al. (2021b) could be illustrated with a complete change in the bacterial community. The fungal community also changes between ethanol addition at room temperature and 10 °C but shows a much smaller degree of modification.

The high production of biomass observed after the addition of ascorbic acid, especially at 10 °C, cannot be explained by the results obtained. The composition of the microbial communities with ascorbic acid are partly the same bacteria and fungi that were present in other samples. The application of this treatment method requires, besides the production of biomass visible in this experiment, the investigation whether and under which conditions soluble microbial products are formed during the denitrification process. To ensure water quality, the COD concentration must also be considered. Our study thus underlines the need for further multi-method investigations with focus on the amount and type of applied organic C under groundwater conditions.

Furthermore, the production of undesirable N compounds such as N_2_O gas needs to be discussed. Laughlin et al. (2009) indicate in their study on N transformation in soils that fungal activity is pronounced when a C substrate is readily available. The respiration measured in their study even shows a dominant activity of fungi, which eventually led to a dominant N_2_O production. N_2_O can be produced not only by fungi but also by bacteria: denitrifying bacteria possess an N_2_O reductase and can further reduce N_2_O to N_2_ (Takaya 2009). However, fungi are assumed to lack N_2_O reductase (Shoun and Tanimoto 1991; Takaya 2009). Nonetheless, N_2_O reductase is sensitive to low pH, which was between 4.1 and 6.0 in the column experiments of our earlier study (Ortmeyer et al. 2021b). Thus, increased N_2_O production can be assumed here as well. In addition, there is the possibility that N_2_O production is also influenced by temperature. This has to remain unanswered for the moment; N gas characterization should be carried out in follow-up studies.

## Conclusions

For a better evaluation of the importance of temperature for enhanced denitrification, the occurrence of bacteria and fungi was investigated in detail in this study. To the best of the authors’ knowledge, this is the first study which analyzes bacterial and fungal communities in the context of enhanced denitrification by addition of organic C, comparing the occurrence between 21.5 °C and 10 °C. While the general occurrence (not the activity) of bacteria is not temperature dependent, fungi preferentially occur at specific temperatures. In addition, at 21.5 °C, the fungi suffer from the addition of organic C and thus partially die, while, at 10 °C, more fungi occur than in the sample without organic C. It can be assumed that the microbiology controls or at least strongly influences the efficiency of individual substrates for enhanced denitrification. We hypothesize a temperature optimum for enhanced denitrification, which differs between the substrates. This temperature optimum presumably depends on the microorganisms which are autochthonous at the respective organic C or under the overall environmental conditions.

Thus, the microbial community is also critically affected by the temperature of the region. Since climate change is expected to increase temperatures in many regions, it can be assumed that microbial communities in groundwater remediation through enhanced denitrification will also be affected by climate change. It will be crucial for future groundwater quality amendment measures to take these connections into account when aiming for an optimal NO_3_^−^ removal efficiency.

## Data Availability

All data generated or analyzed during this study are included in this published article and are available from the European Nucleotide Archive (http://www.ebi.ac.uk/ena/) under study PRJEB42532 and PRJEB52267.
